# A formative evaluation of online information to support abortion access in England, Northern Ireland and the Republic of Ireland

**DOI:** 10.1136/bmjsrh-2018-200086

**Published:** 2018-09-04

**Authors:** Deirdre Niamh Duffy, Claire Pierson, Paul Best

**Affiliations:** 1 Faculty of Health, Psychology and Social Care, Manchester Metropolitan University, Manchester, UK; 2 Department of Politics, University of Liverpool, Liverpool, UK; 3 School of Social Sciences, Education and Social Work, Queens University Belfast, Belfast, UK

**Keywords:** abortion seekers, access to information, access to abortion care, Ireland, England

## Abstract

**Objectives:**

To evaluate web-based information on accessing abortion services retrieved through internet searches in different jurisdictions from the perspective of service users. To provide a formative evaluative mechanism for enabling user-focused design of abortion access information web pages.

**Design:**

Web searches were conducted in three countries—England, Northern Ireland, and the Republic of Ireland—using two search engines in the summer of 2016. Four search terms were used and the first two pages of results were analysed. The perspective of someone seeking abortion services was used. Sources were evaluated using a five-item tool combining user-based indicators identified in other instruments and a question on jurisdictional accuracy.

**Results:**

A total of 619 web pages were retrieved through initial searches, 83 of which related to accessing services; 22 pages were retrieved from the Republic of Ireland, 31 from Northern Ireland, and 30 from England. Fewer than a third (n=31) were judged as good or excellent by the tool. The jurisdictional relevancy of information retrieved varied; almost half of all results in each country provided information that was either inaccurate within or irrelevant to the jurisdiction where the search took place.

**Conclusions:**

If online information is to support abortion access, the circumstances and perspective of the user requires more attention. Designers of abortion information pages online need to ensure that information about access is relevant to the jurisdiction in which users are based.

**Key messages**

- Formative evaluation of online information to support abortion access in the Republic of Ireland, Northern Ireland, and England shows ‘useful’ information is extremely limited.
- Information retrieved by users is not always accurate within the jurisdiction where the search took place. Even ‘good’ web pages are flawed.
- The user needs to be central to the design of web pages.

## Introduction

A recognition of three issues informs this paper: (1) the use of the internet as a source of information about accessing services; (2) the impact of jurisdiction on how services are accessed; (3) the lack of guidance on producing useful internet-based information on how to access abortion services.

The internet is a key source of information related to stigmatised conditions or treatments.^1^ However, web-based access information has been queried and concerns raised— globally and in the UK.^2–4^ In the context of abortion care, usefulness for access is a central question in these discussions. Research in abortion care has highlighted that abortion care seekers using internet searches have difficulties finding information useful for accessing services.^5–7^ WHO argues that poor information is a significant barrier to abortion care and emphasises the need to produce and disseminate clear, accurate information about how and where to access abortion services.^8^ This is rooted in a concern that poor information can delay care, leading to patients accessing abortion at a later stage and at greater risk.

Abortion providers have tried to address these concerns through investing in website design and how service information is presented. However, at the time of writing there is no framework for or guidance on what providers should consider when trying to design web pages that facilitate access effectively. In the absence of such guidance, designers need to rely on existing quality assessment mechanisms such as WebMedQual^9^ or DISCERN.^10^ This reliance is problematic for two reasons. First, these frameworks are quality assessment tools. Their concerns are predominantly issues of scientific accuracy, authorship and rigour. Second, where ‘usefulness for access’ is discussed in these tools, they do not appreciate the peculiarities of access to abortion care. Of particular concern to abortion care access is the accuracy of information within or relevance of information to the location where information searches take place.^8^ Pathways to accessing abortion care are frequently contingent on specific user-related factors (for instance, whether a pregnancy presents a risk to health or whether the pregnancy is the result of rape or incest). Furthermore, pathways can also vary significantly between jurisdictions. In the UK, for example, the policy and legal context of users in Northern Ireland is significantly different to users in England. Whether an information source facilitates access to care is very much contingent on the jurisdiction in which the user is based.

This article highlights that, despite significant investment in designing technically accurate and clearly expressed information resources online, those seeking access to abortion services still face difficulties in accessing relevant, useful information. It illustrates this through the application of a specially-designed formative evaluative instrument to information retrieved through searches in three jurisdictions: the Republic of Ireland, Northern Ireland, and England. In each of these areas there are specific controls on how, when and where an abortion can be accessed and penalties for procuring or providing an abortion outside of these access pathways. Given these differences, information on how to access abortion services may be useful in one jurisdiction but not another. The findings presented in this article provide insight into the challenges faced by abortion services information seekers relying on the internet and draw attention to areas that abortion and health services should be aware of when producing useful web-based information.

Since the research was conducted (in 2016), there have been significant changes in how abortion can be accessed in each country included in the research. In the Republic of Ireland the constitutional ban on abortion was removed by referendum on 26 May 2018. There has also been a campaign to decriminalise abortion in England and Northern Ireland. Women living in Northern Ireland since 2017 no longer have to pay for abortion services in England as private patients. That said, legislation for the procurement and provision of abortion after these changes is yet to be enacted in any of the countries and at the time of publication women still continue to have restricted access to abortion. Furthermore, it is not yet clear what the effect, if any, of these changes will have on abortion information on the internet. The fact that abortion access is currently a core debate in two of the countries included in the research adds a particular timeliness to the paper.

## Methodology

### Objectives

The research aimed to interrogate the usefulness of web-based information on accessing abortion services retrieved through internet searches in different jurisdictions from the perspective of service users. This interrogation would provide a formative evaluative mechanism for enabling user-focused design of abortion access information web pages.

### Research design

The research team evaluated information relevant to accessing abortion services and abortion care on the internet. This includes clinic-based care and self-administration/telemedicine services. (At the time of the research, self-administration/telemedicine services were only available in the two Irish jurisdictions. These services were provided through an activist organisation registered in the Netherlands offering misoprostol to those living in jurisdictions where abortion access is heavily restricted.) The research adopted a formative evaluative approach.^11^ Formative evaluation uses appraisal to identify areas requiring further development within objects of evaluation. In the context of questions of service ‘utility’, its objective is not to provide definitive assessments of usefulness but to enable the effective implementation and design of useful services. As such, our research objectives were to (1) assess the merits of existing sources from a user-centred perspective, and (2) suggest how the utility of online information could be improved.

Formative evaluation is useful not just in the design of services but also as a means of opening up debates on approaches and practices within services. In the context of abortion service information, it draws attention to the problems of placing information on different pages within the website. This is arguably a strategy of organisations in jurisdictions where abortion is criminalised. At the same time, given that research suggests information seekers do not always look beyond the first page of websites, such strategic placement can be problematic. Through formative evaluation, organisations can see the limitations of their approaches to website design and begin to consider how they can facilitate access as much as possible.

### Data collection

The researchers sought to replicate a search pattern similar to that of an individual wishing to access abortion services (therefore an individual who has made the decision to access abortion) using an established methodological approach.^12 13^ Data collection reflected our user-centred approach. We assessed individual web pages retrieved rather than the complete website, an approach resonant with evidence^14^ that health information seekers commonly decide to use a particular service or source based on the first web page accessed.

The three countries—England, Northern Ireland, and the Republic of Ireland—were selected for three reasons. First, when the research was conducted, there was specific legislation regarding the procurement of abortion services in each jurisdiction (this was highly restrictive in Northern Ireland and the Republic of Ireland). Despite ongoing changes in abortion law and policy at the time of publication, it is likely that access to abortion will be contingent on user-related factors with penalties for methods of access outside established pathways. Second, both jurisdictions on the island of Ireland have historic problems with both the dissemination of accurate abortion information and abortion stigma. Third, while England has historically a more liberal approach to abortion access than Irish jurisdictions, abortion access is closely regulated there and the types of abortion service (and user) are very similar. The fact that it is not entirely liberal in its approach to abortion provision and access makes it a good comparator for the Irish jurisdictions.

We used two popular search engines: www.google.co.uk and www.bing.co.uk (.ie in the Republic of Ireland). Searches focused on the relevance to the search query. This meant that web pages retrieved were deemed (by the search engines) to relate to accessing abortion services. These web pages were not always the first page of the individual website. Searches were carried out by the researchers in Northern Ireland (27 July 2016), the Republic of Ireland (24 August 2016) and England (15 September 2016). We used results from the first two pages of search hits as previous studies^2 14^ have found individuals are unlikely to search past even the first page of results.

The researchers spoke to professionals in three organisations—Brook Advisory Clinic Liverpool, the Family Planning Association Belfast, and the Irish Family Planning Association Dublin—to identify appropriate search terms. We chose these organisations based on their extensive knowledge and experience in working with women seeking abortion. We provided an extensive list of possible searches to the organisations and asked each to choose the top three based on their own experience or to suggest alternatives. All organisations agreed that the term ‘termination’ would not be used by an abortion seeker, nor would terms such as ‘crisis pregnancy’. All were in agreement that the following four searches would be the most frequently heard in their professional experience:

1. How to get an abortion
2. Where can I get an abortion?
3. Getting an abortion
4. I need an abortion

### Sample and inclusion

Grounds for inclusion in the study were that identified web pages offered advice or information on accessing abortion services. We applied one screening question: “Is the information about accessing services and/or service-user support?”. Web pages that recorded ‘yes’ were then evaluated. News or commercial web pages and campaigning sites which were not intended to assist access to services were excluded.

### Measurement tool development

In the absence of an existing user-focused evaluative framework for abortion information online, the researchers designed and applied a user-focused five-item tool—the Abortion Service Information Assessment Tool (ASIAT). ASIAT was designed with the user-perspective in mind. Our assessment criteria are resonant with the arguments of Zhang,^15^ who proposed that frameworks for assessing online information should not just include source indicators—such as those identified by WebMedQual^9^ and DISCERN^10^—but also user- and situation-related indicators. This includes the location of the user, the level of user health literacy, age, gender, and requirements (ie, is the search for access to services or for support or information about a specific condition?).

ASIAT combined two issues: (1) whether information seekers would be inclined to use the web page; and (2) whether it would be useful to them. In relation to the first issues, criteria considered in the appraisal included the use of jargon, presence of links to online and offline services, references to existing research, and indications that the web page is monitored by and compliant with professional standards. These criteria reflect the fact that the inclination of information-seekers to use particular online health information is based on heuristics (the design and ‘feel’ of a web page when first encountered) as well as authorship, evidence or the presence of markers of quality compliance.^9 14^ In relation to the second issue, ASIAT assessed jurisdictional relevancy, reflecting the fact that how abortion services are accessed differs across jurisdictions. Descriptions of pathways to services may be useful to information seekers in one jurisdiction but not another.

### ASIAT

ASIAT uses a five-point Likert scale with scores ranging from 5 (lowest) to 25 (highest). Scoring bands were as follows, 5–12 (poor), 13–19 (average) and 20–25 (good). Two researchers conducted preliminary searches and assessments of five web pages independently. They then discussed their assessments and used these to refine the tool. Having refined the tool and updated the scores for the first five results, they then assessed the remaining websites. The final questions used to assess information quality and the criteria informing scoring are outlined in table 1.

**Table 1:** Abortion service information appraisal tool questions and criteria

Question	Criteria	Value range (1–5)
*Is the language used accessible?*	Jargon and technical terms, clarity of expression	Minimum=Jargon heavy, unclear Maximum=No jargon, clearly expressed
*Are links provided to regulated online and offline abortion services?*	Obviousness of links, frequency of links	Minimum=No links Maximum=Links provided clearly and obviously and multiple times
*Are links provided to regulated offline care providers including support groups and information providers?*	Obviousness of links, frequency of links	Minimum=No links Maximum=Links provided clearly and obviously and multiple times
*Is information relevant to the jurisdiction presented clearly?*	References to jurisdictional difference, need for prior knowledge	Minimum=No reference to or recognition of jurisdictional difference Maximum=Clear and obvious references to jurisdictional difference; no prior knowledge required
*Is information quality assured?*	References to/citations from current research, signs that web page is subject to professional standards and compliance	Minimum=No references, citations or signs of professional compliance Maximum=Multiple references to range of evidence/research; indicators that web page is regulated by and compliant with professional standards

### Statistical analysis

Non-parametric tests were used when investigating differences using ordinal scaled data and differences are presented using the median and IQR. All data were analysed using the Statistical Package for Social Sciences (SPSS) version 22.0 (SPSS Inc, Chicago, USA). Means (95% CI) and SD are given where data are normally distributed.

### Patient and public involvement

No patients were involved in this study.

## Results

Overall, 619 web pages were reviewed with 83 (13.4%) meeting the criteria for phase 2 screening. Phase 2 web pages included: 22 (26.5%) from the Republic of Ireland; 31 (37.3%) from Northern Ireland; and 30 (36.1%) from England. Google retrieved 73 (87.9%) of the 83 web pages that met initial screening criteria, whereas Bing retrieved 46 (55.4%).

We identified seven categories of ownership: abortion provider (including abortion pill providers); statutory health provider; campaigning or activist organisation; non-statutory health provider (including sexual and reproductive health information centres); interactive information forums; personal blogs or magazines; and other. The vast majority of web pages (n=52) were authored by abortion and/or health organisations, followed by magazines (n=12) and interactive information forums (n=11). Six pages were owned by activist or campaigning organisations (see online supplementary table 1).

10.1136/bmjsrh-2018-200086.supp1 Supplementary file 1

Of the indicators identified in other measures—language, links with regulated services, content, and authorship—quantitative data revealed a somewhat mixed picture (see online supplementary tables 2–4). While the majority of web pages retrieved used accessible and jargon-free language (n=69), a significant portion (n=23) had no links with regulated services and the content of 32 web pages was either not authored by specialists, unreferenced, or lacked any sign of compliance with professional standards.

The relevancy of information to the jurisdiction where the search took place was problematic in all three jurisdictions (see table 2). Northern Ireland searches returned the highest number of web pages with clearly irrelevant information (n=15/31, 48.4%); this was followed by England (n=14/30, 46.7%) and the Republic of Ireland (n=10/22, 45.5%).

**Table 2:** Information relevant to the jurisdiction

Is information relevant to the jurisdiction presented clearly?	Number of pages (%)
No	39 (47.0)
Yes, but difficult to find and requires prior knowledge	7 (8.4)
Yes, but requires prior knowledge	5 (6.0)
Infrequently	3 (3.6)
Multiple times, requires no prior knowledge	29 (34.9)

In terms of performance against the ASIAT tool, of the 83 web pages assessed, 22 (26.5%) were considered poor, 35 (42.1%) as average, and 26 (31.3%) as good; 6.9% were classified as excellent. Overall web pages rated as good or excellent in terms of usefulness accounted for 4.2% of the entire sample retrieved (n=619) from searches. Mean ASIAT score across all 83 web pages were 16.33 (SD 4.5). No significant differences were found between ASIAT score and jurisdiction (χ^2^=1.573, P=0.455), although web pages retrieved within the Republic of Ireland had the highest overall score (17.3, SD 4.5) with Northern Ireland web pages returning the lowest (15.7, SD 4.5) (see table 3).

**Table 3:** Mean ASIAT score by jurisdiction

Jurisdiction	ASIAT score
England	16.4 (4.9*)
Republic of Ireland	17.3 (4.5*)
Northern Ireland	15.7 (4.5*)
Total	16.33 (4.5*)

*SD.

ASIAT, Abortion Service Information Assessment Tool.

A Kruskal-Wallis test was carried out to examine the difference in ASIAT scores between the seven categories of web page (table 4). This revealed a statistically significant difference between the quality of websites among the seven categories of ownership (χ^2^=25.975, P=0.01). Statutory health provider web pages had the highest median score (19.5, IQR 9.0), with interactive information forums returning the lowest median score (11.0, IQR 4.0).

**Table 4:** ASIAT scores by resource type

Type of resource	ASIAT score*
Abortion provider	17 (7.0)
Statutory health provider	19.5 (9.0)
Campaigning or activist organisation	17 (3.0)
Non-statutory health provider	18 (8.0)
Interactive information forums	11 (4.0)
Personal blogs or magazines	14.5 (5.0)
Other	18 (4.0)

*Median and IQRs presented.

ASIAT, Abortion Service Information Assessment Tool.

Five web pages received the maximum ASIAT score of 25; these included Brook, Positive Options, NHS and NHS direct. Web pages with the lowest ASIAT scores were Quora (7), WikiHow (9), Jezebel, Netmums and Cosmopolitan (10).

As an example of an excellent web page, the Brook website (www.brook.org.uk) scored highly in all categories. Information was written in an accessible language, was provided by reproductive health specialists, multiple links were provided to offline services, information relevant to the jurisdiction was clear and required no prior knowledge, and the site was quality assured with references to standardisation bodies. Interestingly, the NHS website scored lower as it included information irrelevant to either of the Irish legal jurisdictions, limiting its usefulness to users based there. A poor website, for example, Quora, achieved low scores as information was not provided by professionals, it was not relevant to the jurisdiction, no sources or citations were included, and there were no links to regulated offline services.

## Discussion

This research offers insight into what happens when user-based indicators are included in evaluations of abortion service information online. Our research demonstrates that when the relevance and/or accuracy of information about accessing services in the jurisdiction where the search took place is included, the number of useful sources available to information seekers declines. Notably there is a limited difference between jurisdictions with different abortion law and policy, suggesting that a lack of sensitivity to the location of the user is a general problem rather than one restricted to specific jurisdictions. Furthermore, echoing Kunst *et al*’s^4^ observations, the inclusion of jurisdiction as an evaluative criterion indicates that even web pages which have a highly specialist authorship may not be useful to users.

More broadly, ASIAT highlights the challenges facing those seeking information on accessing abortion services. While there is substantial information available through web sources, not all of this information meets the needs of or is useful to abortion seekers. This is a substantial problem in the context of abortion. The limited availability of useful web-based information on services presents a further challenge for those^8 16^ aiming to increase the safety of abortion. Without having accurate information on how to access care, users may delay treatment and have abortions at later stages at greater risk.

There are limitations to this study. ASIAT has not been subjected to peer review in its own right and, as such, the findings can only be interpreted as indicating areas for future investigation. However, the majority of items in ASIAT are informed by questions used in other instruments. Additionally, as a formative evaluative mechanism, ASIAT is intended to highlight areas for development rather than provide conclusive results of quality. A further potential limitation is the fact that the evaluation focuses on the first web page. The usefulness of information on the website as a whole is not included in the assessment. That said, this approach is based on existing research suggesting that internet information seekers frequently only read the first page of websites. Though ASIAT is not comprehensive, it does mimic the actions of internet users.

It is also worth noting that the tool was developed in jurisdictions where the right to information is not limited by law (although in the Republic of Ireland it is closely regulated). This needs to be appreciated when adapting the tool for other jurisdictions where the laws around how and when information about service access is provided are more stringent.

## Conclusion

Regardless of its limitations, this research shows that designers of web-based information need to appreciate user-related and situation-related indicators more actively. Discussions on what counts as good information cannot focus solely on issues of content, credibility and heuristics; the context, particularly the jurisdictional context, in which searches are taking place needs to be considered. ASIAT was designed and applied with two objectives in mind: (1) to focus attention on information seekers in the evaluation of information online; and (2) to offer a mechanism for user-centred formative evaluation of abortion access information online. The tool and the results of the evaluation are noteworthy as they focused explicitly on potential users of abortion services. Both the results of the study and the ASIAT tool are intended to enable web page designers to identify areas for improvement in their own information pages.
