# Supplementary material for: A formative evaluation of online information to support abortion access in England, Northern Ireland and the Republic of Ireland
Source: BMJ Sex Reprod Health. 2018 Sep 4;45(1):32–7. doi: 10.1136/bmjsrh-2018-200086 (PMC6352415; doi:10.1136/bmjsrh-2018-200086)
Supplement: Supplementary file 1 [file bmjsrh-2018-200086supp001.pdf]

Table 1: What type of organisation has produced the webpage?

Type of resource	Number of pages	Percent
Abortion care provider	20	24.1
Statutory health provider	6	7.2
Campaigning/activist organisation	6	7.2
Non-statutory health provider	26	31.3
Interactive information forums	11	13.3
Personal blogs/magazines	12	14.5
Other	2	2.4
Total	83	100.0

Table 2: Is the language accessible? (i.e. is jargon kept to a minimum, is phrasing clear?)

Is the language accessible?	Number of webpages
A little	2
Moderately	9
Reasonably	3
Very	69

Table 3: Links to regulated offline and online services

Are links provided?	Number of webpages	Percentage
No	23	27.7
Yes not clearly	16	19.3
Yes but not frequently or obviously	13	15.7
Yes, clearly and obviously	7	8.4
Yes, clearly, obviously and multiple times	24	28.9

Table 4: Are there indications of quality assurance or evidence-based advice? (i.e. are references to quality assurance/citations provided)

	**Number of webpages**	**Percentage**
No	32	38.6
Infrequent citations, no references to quality assurance	5	6
Some citations/limited references to quality assurance	7	8.4
Clear and obvious citations/no quality assurance references	9	10.8
Clear/obvious/repeated citations and quality assurances references	30	36.1
